# Virulence of *Melissococcus plutonius* and secondary invaders associated with European foulbrood disease of the honey bee

**DOI:** 10.1002/mbo3.649

**Published:** 2018-05-24

**Authors:** Oleg Lewkowski, Silvio Erler

**Affiliations:** ^1^ Institute of Biology, Molecular Ecology Martin‐Luther‐University Halle‐Wittenberg Halle (Saale) Germany

**Keywords:** *Apis mellifera*, brood disease, clonal complex, foulbrood, host–parasite interaction, sequence type

## Abstract

European foulbrood is a globally distributed brood disease affecting honey bees. It may lead to lethal infections of larvae and, in severe cases, even to colony collapse. Lately, a profound genetic and phenotypic diversity was documented for the causative agent *Melissococcus plutonius*. However, experimental work on the impact of diverse *M. plutonius* strains on hosts with different genetic background is completely lacking and the role of secondary invaders is poorly understood. Here, we address these issues and elucidate the impact and interaction of both host and pathogen on one another. Moreover, we try to unravel the role of secondary bacterial invasions in foulbrood‐diseased larvae. We employed in vitro infections with honey bee larvae from queens with different genetic background and three different *M. plutonius* strains. Larvae infection experiments showed host‐dependent survival dynamics although *M. plutonius* strain 49.3 consistently had the highest virulence. This pattern was also reflected in significantly reduced weights of 49.3 strain‐infected larvae compared to the other treatments. No difference was found in groups additionally inoculated with a secondary invader (*Enterococcus faecalis* or *Paenibacillus alvei*) neither in terms of larval survival nor weight. These results suggest that host background contributes markedly to the course of the disease but virulence is mainly dependent on pathogen genotype. Secondary invaders following a *M. plutonius* infection do not increase disease lethality and therefore may just be a colonization of weakened and immunodeficient, or dead larvae.

## 1 INTRODUCTION

European foulbrood (EFB) is a globally distributed brood disease (Ellis & Munn, 2005) mainly infecting early‐stage honey bee larvae of different honey bee species (*Apis mellifera* (Bailey, 1956), *A*. *cerana* (Bailey, 1974), *A*. *laboriosa* (Allen, Ball, & Underwood, 1990)). In the worst cases, EFB can lead to colony death. Diseased larvae are characterized by a change in color from white (via yellow, orange, and brown) to grayish‐black with a foul or sour smell and usually do not reach or complete the pupation stage (reviewed in Forsgren, 2010). The gram‐positive bacterium *Melissococcus plutonius* was identified as the disease‐causing agent (Bailey, 1956, 1957a, 1957b, 1983; White, 1912, 1920) along with some secondary invaders (*Achromobacter eurydice*,* Bacillus pumilus*,* Brevibacillus laterosporus*,* Enterococcus faecalis*,* Paenibacillus alvei*,* Paenibacillus dendritiformis*; Erler, Denner, Bobiş, Forsgren, & Moritz, 2014; Forsgren, 2010; Gaggia et al., 2015). However, the existence of *Achromobacter eurydice* is still controversial (Erler, Lewkowski, Poehlein, & Forsgren, 2018) and the few experimental studies which investigated the impact of secondary invaders focused exclusively the role of *P. alvei* (Giersch, Barchia, & Hornitzky, 2010; Tarr, 1936).

Prevalence of *M. plutonius* is not only high in symptomatic colonies but also in adult bees and larvae of colonies without EFB symptoms located close to EFB‐symptomatic hives (Belloy et al., 2007; Budge et al., 2010; Forsgren, Lundhagen, Imdorf, & Fries, 2005; McKee, Djordjevic, Goodman, & Hornitzky, 2003; Roetschi, Berthoud, Kuhn, & Imdorf, 2008). Even colonies of EFB‐free and (non)symptomatic American foulbrood (caused by *Paenibacillus larvae*) apiaries, far away from EFB‐outbreak regions, have tested positive for *M. plutonius* (Budge et al., 2010; Erban et al., 2017). This means the disease may stay in an enzootic state in apparently healthy colonies (Pinnock & Featherstone, 1984). Transmission between colonies/apiaries occurs via robbing and drifting (White, 1920) where worker bees are the carrier of the bacterium (Belloy et al., 2007). The disease can also be transmitted to artificially reared larvae by using a larval diet inoculated with *M. plutonius* cells (McKee, Goodman, & Hornitzky, 2004).

While early studies assumed low genetic diversity in *M. plutonius,* Allen and Ball (1993) could show potential genetic variability of *M. plutonius* using serological reactivity (polyclonal *M. plutonius* antisera) with heterologous responses reflecting different geographic and host origin of the tested cultures. More recent studies group the approx. 30 different *M. plutonius* sequence types (ST) into three clonal complexes (CC 3, CC12, and CC13) (Budge et al., 2014; Haynes, Helgason, Young, Thwaites, & Budge, 2013; Takamatsu et al., 2014). Sequence types are determined by means of multilocus sequence typing (MLST) and can be detected on both very narrow local scales and widespread across countries (Budge et al., 2014; Haynes et al., 2013; Takamatsu et al., 2014). The different clonal complexes divide *M. plutonius* strains into two subtypes with CC3 and CC13 (including the type strain ST1) containing the typical strains, and CC12 containing the atypical strains (Budge et al., 2014; Haynes et al., 2013; Takamatsu et al., 2014). Typical and atypical (*M. plutonius*‐like) strains can both be isolated from diseased larvae with clinical signs of EFB. However, they differ in their cultural and biochemical characteristics, and their ability to cause disease after laboratory in vitro cultivation (Arai et al., 2012).

Even though all described *M. plutonius* strains (belonging to different clonal complexes) were isolated from naturally diseased honey bee larvae, they seem to differ in virulence to their host. From field pathology data, without experimental testing, CC3 and CC12 are more virulent than CC13, (Budge et al., 2014). Under improved inoculation conditions (potassium‐rich medium/diet) and in vitro larvae infection assays, Nakamura and coworkers proved that CC13 is the least virulent (possibly even avirulent) clonal complex followed by CC3, with CC12 being extremely virulent (Nakamura et al., 2016). Strains of CC13 may lose their infectivity/virulence due to in vitro subcultivation which is a well‐known phenomenon for decades (Bailey, 1956; Bailey & Locher, 1968; Nakamura et al., 2016). Though this loss might be rescued following several in vivo passages in honey bee worker larvae (Bailey, 1963).

Most of the studies focusing on honey bee host–parasite (pathogen) interactions studied pathogen genotypic effects (Genersch, Ashiralieva, & Fries, 2005) rather than host–parasite/pathogen genotypic interactions (Evison et al., 2013). For bacterial bee diseases in particular, host genotypic effects are usually disregarded. Here, we investigate the virulence of several *M. plutonius* isolates (belonging to CC3 and CC13 complex) on different host genetic backgrounds using standard in vitro larvae rearing. Furthermore, we tested the most virulent *M. plutonius* strain, in combination with two secondary invaders (*P. alvei* and *E. faecalis*), for their putative additive pathogenic effects.

## 2 MATERIAL AND METHODS

### 2.1 Ethics statement

Endangered or protected species were not used in this study. Experiments and observations conform with the laws of Germany in relation to animal protection. No specific ethics certification is required for this research.

### 2.2 Impact of host background

#### 2.2.1 Larvae grafting and in vitro rearing

Honey bee larvae (*Apis mellifera*) were grafted, reared in vitro and received worker larval diet as described in Crailsheim et al. (2013). To test for variance in *M. plutonius* CC‐type virulence and colony effects, hatched larvae were grafted from two different colonies (A— queen of Czech‐German origin, B—queen of French‐German origin), following queen caging for 24 hr, and assigned randomly to one of the four following treatment groups per colony. Treatment group 1: control (uninfected), and three groups (group 2–4)—larvae infected with *M. plutonius* strains 49.3 (Switzerland/Jenaz), 119 (Switzerland/Köniz) or 4–127 (Sweden). Both colonies were treated against *Varroa destructor* using Bayvarol stripes (Bayer) according to the manufacturer recommendations and were checked regularly for clinical signs of common bee diseases. No symptoms, such as bees having crippled wings (DWV) or diarrhea (*Nosema* sp.) were observed, indicating a similar healthy status of both colonies.

#### 2.2.2 Bacteria cultivation and infection

*M. plutonius* strains 49.3 (ST 3, CC3), 119 (ST 20, CC13), 4–127 (ST1, CC13), and LMG 20360 (ST1, CC13) were cultivated in a liquid medium consisting of 5 g/L yeast extract, 2 g/L sucrose, 10 g/L glucose, 6.75 g/L KH_2_PO_4_, 6.75 g/L K_2_HPO_4_, 1 g/L L‐cysteine hydrochloride, and 5 g/L homogenized drone pupae (white eye), adjusted to pH 6.6 with KOH and incubated at 35°C with 10% CO_2_. Cultivation success and bacteria species was verified by sequencing according to Erler et al. (2014).

Worker bee larvae were grafted on a 5 µl day 1 diet (Crailsheim et al., 2013) and subsequently fed with 5 μl of a diet‐inoculum mix (19:1) containing one of the respective bacterial strain cultures or sterile medium (controls). The absorbance of the bacterial solutions was adjusted to 0.3 (OD_600 nm_) beforehand. From day 2 postgrafting till day 6 postgrafting, bee larvae received a standard diet following Crailsheim et al. (2013).

To estimate the effective colony‐forming units (CFUs) fed to the larvae, 20 μl of diluted inoculum (diet with bacteria) (1:50, 1:1,000, 1:10,000), as well as noninoculated food, were plated on solid medium plates (1.5% agar) according to Forsgren, Budge, Charrière, and Hornitzky (2013). CFUs were counted after 3 days incubation (35°C, 10% CO_2_) and are given per ml culture medium.

#### 2.2.3 Larval survival, weight, and relative *M. plutonius* infection intensity

Larval survival was monitored on a daily basis until pupation of all larvae of the noninfected control groups. Pupation means that each larva had a clearly recognizable head–thorax–abdomen structure and six legs. Lastly, every individual that did not fully pupate the same day as the control bees was counted as dead. Larvae of all groups that died on day 1 postgrafting were excluded from the survival analysis due to potential grafting errors (Crailsheim et al., 2013). Larval weight was measured on day seven for all larvae of all groups, before placing them into new plates (covered with cellulose) for pupation (Crailsheim et al., 2013).

Larval infection intensity was measured from 8 to 12 white larvae per treatment group (minimum 4 larvae per group and for at least 2 grafting events), randomly selected on day 10 ± 1 postgrafting. White larvae which did not defecate were selected to get a consistent picture of *M. plutonius* replication by excluding extremes (e.g., brown, gray, and black rotten larvae). DNA isolation was performed following standard phenol–chloroform–isoamyl alcohol (25:24:1) protocol. Initially larvae were homogenized in 400 μl extraction buffer pH 8.0 (100 mmol/L Tris/HCl, 100 mmol/L NaCl, 10 mmol/L EDTA, 1 mmol/L SDS) with 1 μl Tween20, and incubated for 5 min on ice. At the final step of the extraction procedure the dried DNA pellet was resuspended in 20–30 μl elution buffer (10 mmol/L Tris pH 7.4, 1 mmol/L EDTA pH 8.0). DNA quality and quantity were determined on a NanoDrop 1000 (Thermo Fisher Scientific). Samples with more than 1 μg/μl were diluted 1:50 and samples below 1 μg/μl 1:20 with DEPC water prior to *M. plutonius* quantification (qPCR) using the CFX Connect Real‐Time PCR Detection System (Bio‐Rad), SensiMix‐SYBR No‐ROX Kit (Bioline), and *M. plutonius*‐specific primers (EFB‐primer; Budge et al., 2010). Relative *M. plutonius* bacterial load per larvae (Forsgren et al., 2013) was estimated by normalizing bacterial load to the amount of honey bee mitochondrial DNA (COI‐primer) as recommend by Behrens, Forsgren, Fries, and Moritz (2010). The same qPCR protocol was used for each 10 μl reaction (including 1 μl diluted DNA) and one of the two primer pairs (forward and reverse, each with 0.3 μmol/L): initial denaturation at 95°C for 10 min, 40 cycles of 95°C for 15 s, 60°C for 30 s and 72°C for 30 s. The melting curve of the amplicons was measured from 50°C to 98°C, every 5 s with 1°C increment. Each sample was analyzed in duplicates and repeated if between replicate difference (delta C_q_) was higher than 0.5. PCR efficiency for each primer pair was estimated using DNA dilutions: PCR_eff_ (EFB‐primer) = 1.93, PCR_eff_ (COI‐primer) = 1.89. Finally, data were collected for the two colonies, four treatment groups and for each combination of larvae from two to three grafting events. Details on exact sample sizes are summarized in Table S1.

#### 2.2.4 Statistics

All statistical analyses were performed using R (ver. 3.3.2). Larval survival was evaluated by means of Kaplan–Meier survival analysis with subsequent log‐rank tests, for colony and treatment effects.

Data for larval weight and relative *M. plutonius* infection intensity were tested for deviation from a normal distribution by Kolmogorov–Smirnov tests. As a normal distribution could not be confirmed for both, nonparametric tests were used to test for significant differences between colonies (Mann–Whitney *U* test) and treatment groups (Kruskal–Wallis ANOVA). *P*‐values were adjusted for multiple testing using Bonferroni correction.

### 2.3 Impact of coinfection

#### 2.3.1 Larvae grafting and in vitro rearing

In a second set up we tested the impact of secondary invaders on larval survival and weight. Grafting, rearing, and infection of larvae were performed as described above using larvae from a strong colony (colony C—queen of German origin) of the university stock. Experiments were run in four replicates. The first replicate contained control and *M. plutonius* strain 49.3‐infected individuals while the following three replicates additionally included treatments with *M. plutonius* strain 49.3 combined with *Enterococcus faecalis* or *Paenibacillus alvei*.

#### 2.3.2 Bacteria cultivation and infection

*M. plutonius* strain 49.3 was cultivated and applied as described for the first experiment. On day 3 postinfection with *M. plutonius* strain 49.3, the larvae received 30 μl of standard larval diet (Crailsheim et al., 2013) mixed with vegetative cells of a secondary invader (*E. faecalis* or *P. alvei*, approx. 6 × 10^4^ CFUs per larvae for each bacteria species) or sterile medium (control—just *M. plutonius* treatment). *Enterococcus faecalis* (LMG 7937) and *Paenibacillus alvei* (LMG 13253) were provided by BCCM/LMG Bacteria Collection (Ghent University, Ghent, Belgium) and grown in specific medium as described elsewhere (Erler et al., 2014). The absorbance of both bacterial cultures was adjusted to 0.3 (OD_600 nm_) and bacteria were fed to larvae as described above (approx. 6 × 10^4^ CFUs per larvae). CFUs for *E. faecalis* and *P. alvei* finally consumed by the larvae were determined with 20 μl diluted inoculum (infective diet) (*E. faecalis*: 1:10,000, 1:100,000; *P. alvei*: 1:1,000, 1:10,000) and plated on specific solid medium (1.5% agar), as well as noninoculated food. The colonies were counted after 1 day of incubation at 35°C and are given per ml of culture medium (Table S2).

#### 2.3.3 Larval survival and weight

Larval survival was determined as described above but the weight was measured on day eight to account for the growth of the secondary invaders.

#### 2.3.4 Statistics

Survival analysis was performed with a nested Cox regression mixed‐effects model (coxme package in R) fitted by maximum likelihood with treatment as fixed effect and replicate as random effect (treatment groups nested in replicates) to account for the variance in the replicates, as replicates 1 and 3 differed significantly from 2 and 4 but not from each other respectively.

Larval weights were analyzed as described previously.

## 3 RESULTS

Before examining variable virulence of several natural *M. plutonius* isolates, we examined the in vitro virulence of a commercially available *M. plutonius* strain (LMG 20360, ST1, CC13, the only one commercially available) in larvae from colonies of different origin (colony A and B). However, neither a significant impact on larval weight on day 7 (MW*U* test: W = 2447.5, *p = *.14, Table S3) nor on larval mortality (Kaplan–Meier survival analysis with log‐rank test, *p *=* *.32, Figure S1) was observed in *M. plutonius* exposed larvae, but a significant weight difference between larvae of different origin (MW*U* test: W = 2428.5, *p < *.0001, Table S3) with larvae of colony B being lighter compared to colony A.

### 3.1 Impact of host background

**Figure 1:**
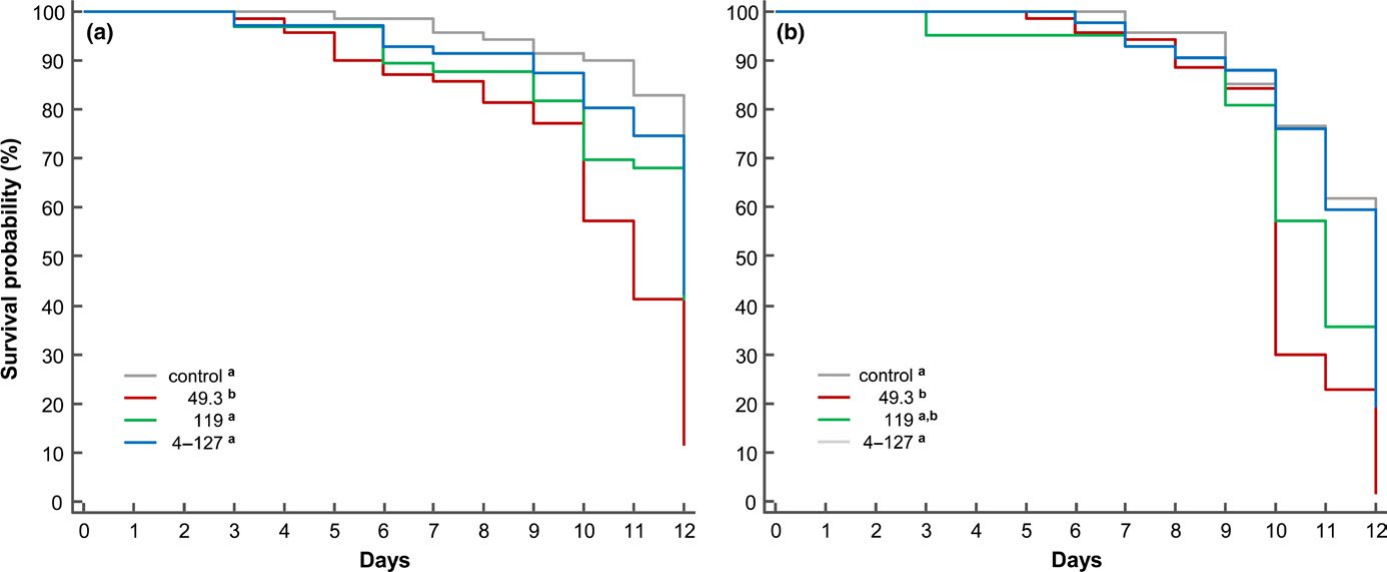
Larval survival over 12 days (starting from day 1—grafting) for control groups and larvae infected with *M. plutonius* (strain 49.3, 119 and 4‐127) from two unrelated colonies (a) colony A, (b) colony B. Different superscript letters in the legend for treatment groups show significant differences in larval survival, following Kaplan–Meier survival analyses with Bonferroni adjusted posthoc log‐rank tests (*p *<* *.016)

Comparing overall larval survival of the noninfected control groups for colony A and B revealed a reduced survival rate of larvae grafted from colony B (Kaplan–Meier survival: *Z *=* *3.24, *p *=* *.0012). The same effect was observed when pooling all larvae (across all treatment groups) of colony A and B (*Z *=* *5.18, *p *<* *.0001), however, with the exception that mortality at the larval stage (day 1–6 postgrafting, Figure 1) was lower for colony B than for colony A. Nevertheless, virulence of the three different *M. plutonius* strains tested showed to be similar for both colonies. Strain 49.3 (CC3) was the most virulent leading to mortality rates higher than 85% at day 12 postgrafting and differing from all other groups (log‐rank tests, *p *<* *.001) (Figure 1). No difference was measured between the noninfected control groups and larvae infected with strain 4–127 (CC13) (log‐rank tests, *p *>* *.05) (Figure 1). Variable results for strain virulence have been observed for strain 119 (CC13), showing no difference from controls and larvae infected with 4–127 for colony A and B (log‐rank tests, *p *>* *.05). For colony A, the difference in mortality was highly significant between strains 119 and 49.3 (log‐rank test, *p *<* *.001) but not for colony B (log‐rank test, *p *=* *.017, Bonferroni adjusted significance level: *p *<* *.016) (Figure 1). This borderline nonsignificant difference for colony B, following *p*‐value adjustment, indicates potential variable virulence for strain 119 depending on host genetic background and/or general health conditions of the tested colonies.

Larval weight was again lower for control larvae grafted from colony B (72.21 mg ± 31.96, mean ± *SD*) compared to colony A (101.25 ± 25.73) (MW*U* test: *Z *=* *4.59, *p *<* *.0001) as well as overall treatment groups (MW*U* test: *Z *=* *7.44, *p *<* *.0001) (Table 1). Weight differences between treatment groups and strains showed similar patterns for colonies A and B. All groups (control, strain 119 and 4–127) did not differ from each other but from 49.3 which had the lightest larvae (colony A: 62.92 mg ± 30.2, colony B: 46.99 mg ± 19.14) (Kruskal–Wallis ANOVA: *H*_colony A_ = 48.76, *H*_colony B_ = 31.84, posthoc multiple comparisons: *p *<* *.0001 for both) (Table 1). The least significant difference was again observed for colony B with larvae infected with the strains 49.3 and 119 (*p *=* *.006, all other comparisons: *p *<* *.00015) (Table 1).

**Table 1:** Larval weight (mean ± *SD*) on day 7 (day 6 postinfection) for control groups (uninfected) and larvae infected with *M. plutonius* (strain 49.3, 119 and 4–127) from two unrelated colonies (A and B)

Colony	Treatment	Weight (mg)	*N*
A	Control	101.3 ± 25.7^a^	65
	49.3	62.9 ± 30.2^b^	59
	119	92.9 ± 33.8^a^	58
	4–127	94.7 ± 28.0^a^	65
	Total		247
B	Control	72.2 ± 32.0^a^	44
	49.3	47.0 ± 19.1^b^	76
	119	72.8 ± 41.3^a^	38
	4–127	81.1 ± 37.3^a^	39
	Total		197

Different superscript letters show significant differences between treatment groups.

The number of colony‐forming units (CFUs) per larva used for infection was different for strains 4–127 in comparison with 49.3 and 119 (One‐way ANOVA with log‐transformed data: *F *=* *8.39, *p *=* *.002, Bonferroni posthoc tests: 4–127 *vs*. 49.3: *p *=* *.048, 4–127 *vs*. 119: *p *=* *.001) but not between the last two strains (Bonferroni posthoc test, *p *>* *.05) (Table S1). Irrespective of the dilution used to estimate CFUs, 4–127 always grew in much higher CFU number with a smaller size (colony diameter) than for the other two strains (49.3 and 119); at least, on agar plates in the CO_2_‐incubator.

No differences were detected for the relative *M. plutonius* infection intensity per larvae neither between colonies (means and 95% confidence intervals; colony A: 0.0070, CI 0.0038–0.0103; colony B: 0.0053, CI 0.0026–0.0081; MW*U* test: *Z *=* *0.77, *p *>* *.05) nor between strains (means and 95% confidence intervals; 49.3: 0.0038, CI 0.0025–0.0051; 119: 0.0068, CI 0.0033–0.0102; 4–127: 0.0088, CI 0.0029–0.0147; Kruskal–Wallis ANOVA: *H *=* *2.03, *p *>* *.05). All infected larvae tested were positive for *M. plutonius* (C_q_ values across colonies and strains: 21.05 ± 2.05, mean ± *SD*) and all noninfected control larvae were negative.

### 3.2 Impact of co‐infection

**Figure 2:**
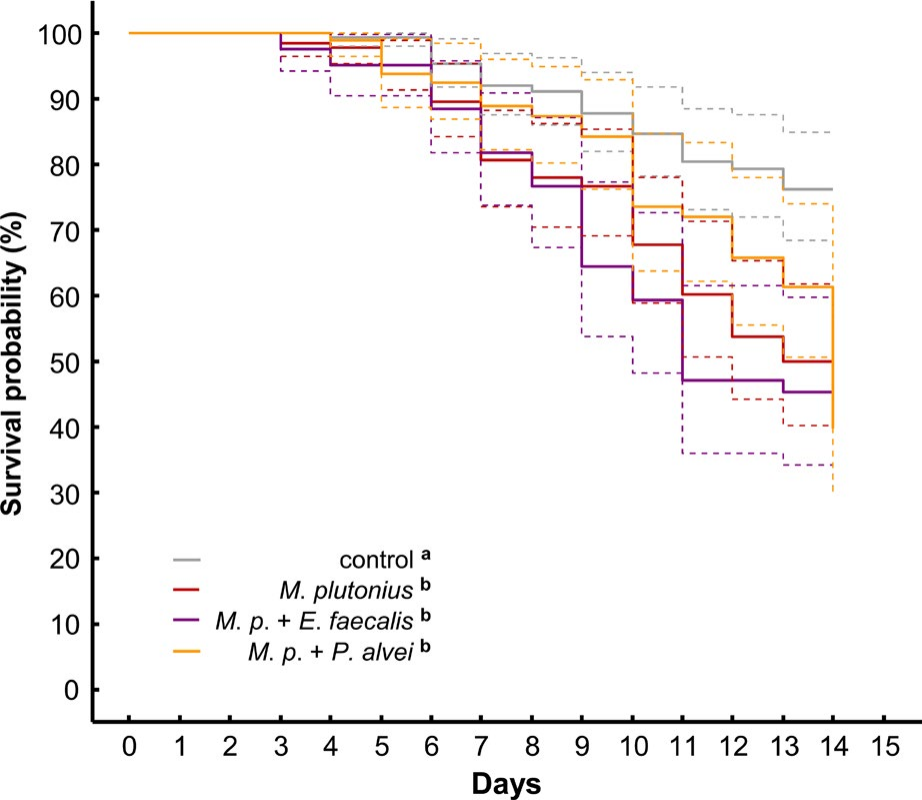
Larval survival over 14 days (starting from day 1—grafting) for control group and larvae infected with *M. plutonius* strain 49.3, *M. plutonius* + *E. faecalis* or *M. plutonius* + *P. alvei*. Different superscript letters in the legend for treatment groups show significant differences in larval survival, following nested Cox regression mixed‐effects model analyses with Bonferroni adjusted posthoc log‐rank tests. (Dashed lines: 95% confidence intervals)

Although, all infection treatment groups (irrespective of a secondary bacterium combination or solely *M. plutonius*) had significantly higher mortality rates compared to the noninfected controls (Cox regression mixed‐effects model: *p < *.001), there was no significant difference between the treatment with *M. plutonius* only (M) and the treatments with an additional infection (*E. faecalis*: ME, *P. alvei:* MP) (log‐rank test pairwise comparisons, Bonferroni adjusted: M‐ME *p *=* *.38, M‐MP *p = *.91, MP‐ME *p *=* *.38, Figure 2).

For the larval weights we observed a similar outcome. Infection treatments resulted in significantly lower larval weight compared to controls (Kruskal–Wallis ANOVA: *H *=* *16.88, *p *=* *.0007; posthoc multiple comparisons, Bonferroni adjusted: M‐C *p = *.004, ME‐C *p = *.02, MP‐C *p = *.02, Table 2) with approx. 15% mean weight reduction in infected individuals (Table 2). Consistently, no weight differences were detected between infected groups (posthoc multiple comparisons, Bonferroni adjusted: M‐ME, M‐MP, MP‐ME *p *>* *.05, Table 2).

**Table 2:** Larval weight (mean ± *SD*) on day 8 (day 7 postinfection with *M. plutonius* strain 49.3) for control groups (uninfected) and larvae infected with *M. plutonius* strain 49.3 only or *M. plutonius* and secondary invaders *E. faecalis* or *P. alvei* on day 4 (day 3 postinfection with *M. plutonius*)

Treatment	Weight (mg)	*N*
Control	134.7 **±** 24.3^a^	83
*M. p*.	113.7 **±** 40.0^b^	62
*M. p*. + *E. f*.*	115.4 **±** 37.7^b^	40
*M. p. *+ *P. a*.*	113.7 **±** 40.6^b^	42
Total		227

*M. p. treatment on day 1.

Different superscript letters show significant differences between treatment groups.

The CFUs per larva used for infection varied between replicates, especially in *E. faecalis* treatments (Table S2), although it did not correlate with larval mortality or weight, neither for *M. plutonius* infected larvae nor larvae treated with an additional infection.

## 4 DISCUSSION

In this study, we observed variation in virulence for four different *M. plutonius* strains and a divergent response of hosts depending on genetic background. Here, the determinative host trait for the resulting variation of pathogen impact seems to be the developmental differences (specifically body size) of the honey bee larvae. Host body size and developmental speed may be crucial factors in host–pathogen interactions where the pathogen may be obliged to multiply as fast as possible to overcome clearance by the host (Cable, Enquist, & Moses, 2007). This might particularly be critical for a pathogen infecting a host via the oral–fecal route. In the case of the honey bee larvae, clearance refers to defecation before pupation. An alternative hypothesis explaining variance in larval susceptibility might be enhanced defense mechanisms of specific host genotypes (McKee et al., 2004).

The observed weight differences of infected larvae of different origin are negatively correlated with mortality (Pearson correlation: *n* = 8, *r* = −.97, *p < *.0001). At larval stage, the mortality of the lighter phenotype (colony B) was lower compared to the heavier one (colony A) (Figure 1). That lower weight appears to be advantageous, is presumably due to reduced multiplication rates of the pathogen. However, in the pupal stage, the apparently slower growth rate of larvae from colony B phenotype led to a reduced nutrient supply during metamorphosis and ultimately resulted in a higher mortality of pupae. Malnutrition caused by *M. plutonius* infections, leading to reduced larval and pupal weight, was already discussed several times (Bailey, 1959, 1960; Nakamura et al., 2016). However, at this point no inferences could be made on the cause(s) leading to malnutrition, either due to host–pathogen competition for nutrients or due to less nutrients feed to the larvae in the colony or both.

Mortality of the different honey bee strains used for larval infection was strongly influenced by the host background and sequence type of the *M. plutonius* strain. With the limited number of different sequence types tested per clonal complex (CC3 and CC13), we cannot make any final conclusion on virulence diversity of the different clonal complexes. The most interesting case concerning virulence was strain 119 which can cause variable mortality depending on the host background (Figure 1). In a previous study, *M. plutonius* strain 119 showed high virulence leading to mortality rates of 70% in 2 weeks (Riessberger‐Gallé, Hernández‐López, Rechberger, Crailsheim, & Schuehly, 2016). This corresponds to the higher virulence in colony B of the current study (Figure 1).

Future studies should investigate additional representative pathogen genotypes of the different clonal complexes or genetically modified strains to understand the connection of *M. plutonius* genotype and virulence. More importantly, the degree of virulence and the impact of mixed infections with typical and atypical *M. plutonius* strains is not well understood and adds to a far more complicated picture. In the current study, we exclusively tested typical strains. From previous studies, we know that both *M. plutonius* strain types can be detected by duplex PCR and cultivation in the same larvae samples (Arai et al., 2014) and several strains (sequence types) might participate in single EFB outbreaks of the same apiary, which, at least in Japan, might be common (Takamatsu et al., 2014).

The results of Giersch et al. (2010) for *M. plutonius* infections and a disease‐specific secondary invader, could not be confirmed in this study. They found higher mortality and infection rates in *M. plutonius*‐ and *P. alvei*‐inoculated larvae and observed much stronger typical symptoms compared to only *M. plutonius*‐infected larvae. By using a comparative infection assay (time of infections) here, some major differences have to be mentioned. Giersch et al. (2010) used different methods determining bacteria/spore concentrations and CFUs, a different feeding protocol, spores instead of vegetative cells for *P. alvei*, and genotypes of *M. plutonius* (putatively CC13, ST4) and *P. alvei* strains were of Australian origin. At least for adult bees, a recent study showed that single infection with *E. faecalis* does not decrease mortality (Dickel, Münch, Amdam, Mappes, & Freitak, 2018). This may confirm that we did not find any additive mortality by coinfecting larvae, as previously mentioned by Bailey (1963). However, this is highly speculative and has to be verified by infection experiments with healthy larvae and *E. faecalis* or *P. alvei* only.

Although, during the current study there was an EFB‐typical foul smell perceptible in diseased larvae of the *M. plutonius*/*P. alvei* treatment as well as in *M. plutonius*/*E. faecalis* treatment, no differences for larval mortality or weight were observed for the different infection treatments. Similar to virulence of *M. plutonius* there might also be strain specificity in the secondary invaders like *P. alvei* and *E. faecalis* and they may even originate from the larval gut microbiota (commensals) (Overstreet & Lotz, 2016). So far, we have no conclusive explanation for the different outcome of both infection studies (Giersch et al., 2010; present study), except for the bacterial strains and host genetic background. Both experiments shown here, revealed that infection dose might not play a central role for the outcome of the infection, once a minimum threshold (not determined) has been reached. Similarly, variance in CFUs for *M. plutonius* and secondary invader correlated neither with mortality, weight, nor with infection intensity.

For artificial infections, the quality of the larval food (batch of royal jelly) seems to be of high importance for infection success (Giersch et al., 2010). *M. plutonius* cell numbers can be significantly reduced by a highly antimicrobial royal jelly water extract, based on major royal jelly protein 1, fatty acids and other substances (Vezeteu, Bobiş, Moritz, & Buttstedt, 2017; and references therein). We did not examine the resistance of *M. plutonius* strains to larval food used in this study. However, a recent study showed that clonal complex affiliation and number of days precultured are major factors explaining resistance for in vitro growth in medium containing royal jelly or 10‐hydroxy‐2‐decenoic‐acid (10‐HDA) (Takamatsu, Osawa, Nakamura, Yoshiyama, & Okura, 2017).

Another point of *M. plutonius* pathogenicity, not analyzed in the current study, is the delay of symptoms under non‐natural conditions. Artificially infected colonies show a disease symptom delay of up to 12 days under natural conditions (Bailey, 1957b). Laboratory artificial infections of bee larvae also lead to a delay in defecation and subsequent, if any, pupation (McKee et al., 2004). The same study described that some larvae even survived the infection and reached pupation. This might be the result of *M. plutonius* variability of multiplication speed and virulence or host susceptibility.

## 5 CONCLUSION

The environment of the colony has to be mentioned as an important factor which profoundly affects the course of the disease. However, the details are nearly unknown (Bailey & Locher, 1968; White, 1920). Former studies on *M. plutonius* epidemiology showed that visible EFB‐symptoms can disappear spontaneously from infected colonies after an epidemic peak (about June in the northern hemisphere) which appears to be unrelated to changes in the susceptibility of the larvae as they still can be infected in vitro (Bailey, 1959, 1960; Burnside, 1938; White, 1920).

To get a deeper understanding of the pathogenicity of *M. plutonius* and the nature of EFB it is imperative to study the natural epidemiology of the disease in combination with in vitro and in vivo assays investigating the growth and virulence of the diverse *M. plutonius* types (typical vs. atypical), clonal complexes and genotypes (sequence types). From the current and all previous studies, the whole infection process seems to be a complex interaction of the honey bees’ and *M. plutonius* genotype, in combination with several secondary invaders.

## CONFLICT OF INTEREST

The authors declared no potential conflict of interest.
