# Supplementary material for: Virulence of Melissococcus plutonius and secondary invaders associated with European foulbrood disease of the honey bee
Source: Microbiologyopen. 2018 May 24;8(3):e00649. doi: 10.1002/mbo3.649 (PMC6436434; doi:10.1002/mbo3.649)

<u>**Supplementary material**</u>

Virulence of *Melissococcus plutonius* and secondary invaders associated with European foulbrood disease of the honey bee

Oleg Lewkowski, Silvio Erler

Table S1: Sample overview (number of individuals) per colony for each assay of experiment 1 – 'Impact of host background': survival, larval weight on day 7, relative *M. plutonius* load per larvae; and CFUs used for infection for each treatment group (mean ± SE).

Colony	Treatment	Survival	Larval weight	*M. plutonius* load	CFUs per ml	CFU per larvae
A	control	70	65	8	0	0
	49.3	70	59	12	$4.8\times10^6 \pm 1.6\times10^6$	1206 ± 398
	119	66	57	12	$3.2\times10^6 \pm 1.5\times10^6$	797 ± 380
	4-127	71	65	12	$7.0\times10^7 \pm 3.4\times10^7$	17512 ± 8424
	total	277	246	44		
B	control	47	44	8	0	0
	49.3	84	76	12	$4.8\times10^6 \pm 1.6\times10^6$	1206 ± 398
	119	42	38	8	$3.2\times10^6 \pm 1.5\times10^6$	797 ± 380
	4-127	42	39	8	$7.0\times10^7 \pm 3.4\times10^7$	17512 ± 8424
	total	215	197	36		

Table S2: Sample overview (number of individuals) for each assay of experiment 2 – 'Impact of co-infection': survival, larval weight on day 8; and CFUs used for infection for each treatment group (mean ± SE).

Treatment	Survival	Larval weight	CFUs per ml	CFU per larvae
control	73	83	0	0
M. p.	59	62	$1.4\times10^8 \pm 6.3\times10^7$	34605 ± 15654
*E. f.**	65	40	$1.1\times10^9 \pm 5.2\times10^8$	$2.7\times10^5 \pm 1.3\times10^5$
*P. a.**	69	42	$3.4\times10^7 \pm 1.4\times10^7$	8387 ± 3474
total	266	227		

* *M. p.* treatment on day 1

Table S3: Larval weight (mean ± SD) on day 8 (day 7 post-infection) from the preliminary infection experiment. Controls and *M. plutonius* (strain LMG 20360, ST1, CC13) inoculated larvae.

Colony	Treatment	Weight (mg)	N
A	control	139.6 ± 23.2	23
	LMG 20360	148.1 ± 20.3	66
	total		89
B	control	124.4 ± 33.3	23
	LMG 20360	134.7 ± 21.0	64
	total		87

Fig. S1. Larval survival in preliminary infection experiment (*M. plutonius* strain LMG 20360, ST1, CC13) with colony A and colony B (cox regression model: $P > 0.05$).

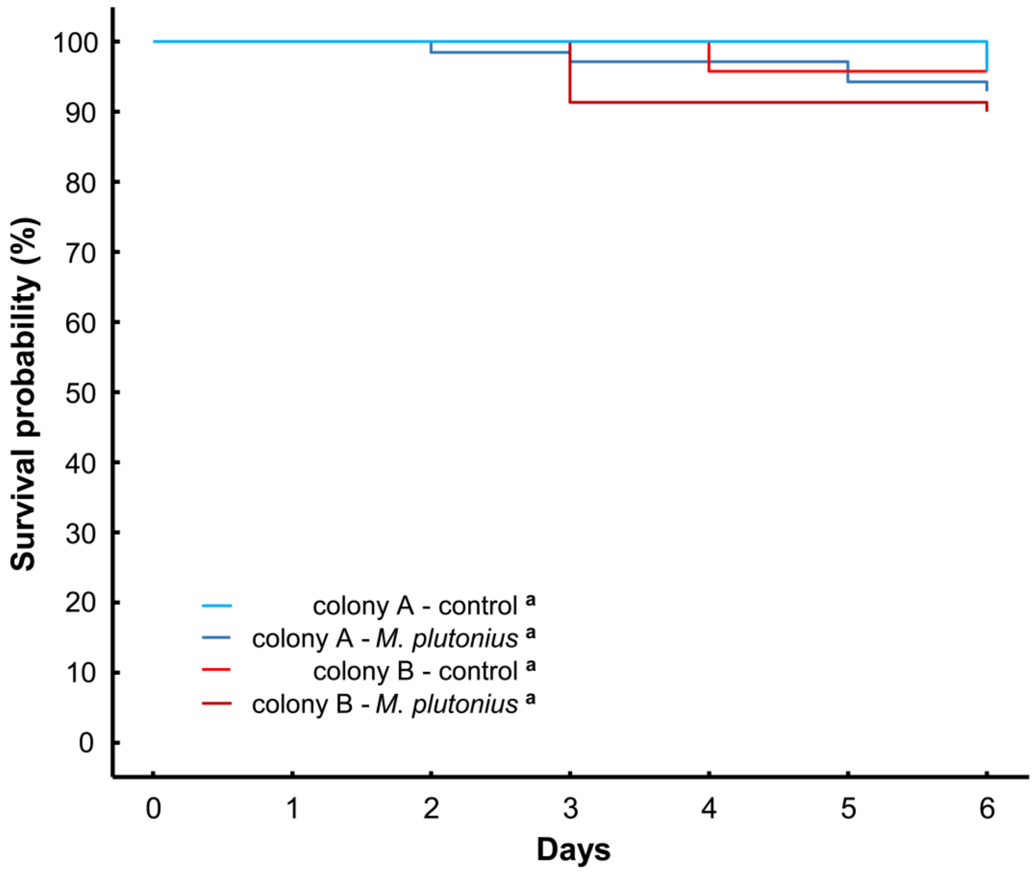

Supplement: Supplementary file 1 [file MBO3-8-e00649-s001.pdf]
